# CFTR corrector C17 rescues defective SERCA1 in bovine pseudomyotonia: a potential therapy for Brody myopathy

**DOI:** 10.1093/hmg/ddaf142

**Published:** 2025-11-06

**Authors:** Eylem E Akyürek, Joana Gonçalves Pontes Jacinto, Silvia Iori, Elisa Bianchini, Marilena Bolcato, Roberta Costa, Mery Giantin, Marcello Carotti, Mauro Dacasto, Giovanna Cenacchi, Dorianna Sandonà, Arcangelo Gentile, Roberta Sacchetto

**Affiliations:** Department of Comparative Biomedicine and Food Science, University of Padova, viale dell'Università 16, Legnaro 35020, Padova, Italy; Clinic for Ruminants, Vetsuisse Faculty, University of Bern, Länggassstrasse 120, Bern 3012 Switzerland; Institute of Genetics, Vetsuisse Faculty, University of Bern, Bremgartenstrasse 109A, Bern 3012 Switzerland; Department of Comparative Biomedicine and Food Science, University of Padova, viale dell'Università 16, Legnaro 35020, Padova, Italy; Department of Biomedical Sciences, University of Padova, via Ugo Bassi 58/b, Padova 35131, Italy; Evotec, via Alessandro Fleming 4, Verona 37135, Italy; Department of Veterinary Medical Sciences, University of Bologna, via Tolara di Sopra 43, Ozzano Emilia 40064, Bologna, Italy; Department of Biomedical and Neuromotor Sciences, University of Bologna, Via Massarenti 9, Bologna 40126, Italy; Department of Comparative Biomedicine and Food Science, University of Padova, viale dell'Università 16, Legnaro 35020, Padova, Italy; Department of Biomedical Sciences, University of Padova, via Ugo Bassi 58/b, Padova 35131, Italy; Department of Comparative Biomedicine and Food Science, University of Padova, viale dell'Università 16, Legnaro 35020, Padova, Italy; Department of Biomedical and Neuromotor Sciences, University of Bologna, Via Massarenti 9, Bologna 40126, Italy; Department of Biomedical Sciences, University of Padova, via Ugo Bassi 58/b, Padova 35131, Italy; Department of Veterinary Medical Sciences, University of Bologna, via Tolara di Sopra 43, Ozzano Emilia 40064, Bologna, Italy; Department of Comparative Biomedicine and Food Science, University of Padova, viale dell'Università 16, Legnaro 35020, Padova, Italy

**Keywords:** human Brody disease, Sarco/Endoplasmic reticulum Ca2 + -ATPase, isoform 1 (SERCA1), cystic fibrosis transmembrane regulator (CFTR) correctors, bovine congenital pseudomyotonia

## Abstract

Brody myopathy is an ultra-rare autosomal recessive inherited disorder that impairs skeletal muscle function in humans. It is caused by deficiency of the Sarco(Endo)plasmic reticulum Ca^2+^-ATPase isoform1 (SERCA1), arising from defects, mainly missense mutations, in the ATP2A1 gene. At present, neither specific therapy, nor mouse model exists for Brody myopathy. Bovine pseudomyotonia (PMT) is a very rare skeletal muscle disorder. As Brody myopathy, it is an autosomal recessive inherited disorder caused by missense variants in the *atp2a1* gene. Most mutations generate proteins corrupted in proper folding that although catalytically active, were ubiquitinated and prematurely degraded by the ubiquitin-proteasome system, thus sharing with Cystic Fibrosis the same pathogenetic mechanism. Bovine PMT, despite unconventional, is currently the unique mammalian model of Brody disease. In this study, we show that CFTR correctors, particularly C17, successfully rescue SERCA1 mutants both *in vitro* and *in vivo* models. Our findings suggest that CFTR correctors may be a potential innovative pharmacological approach addressing Brody patients in which mutated SERCA1 retains its activity.

## Graphical Abstract

**Graphical Abstract:**
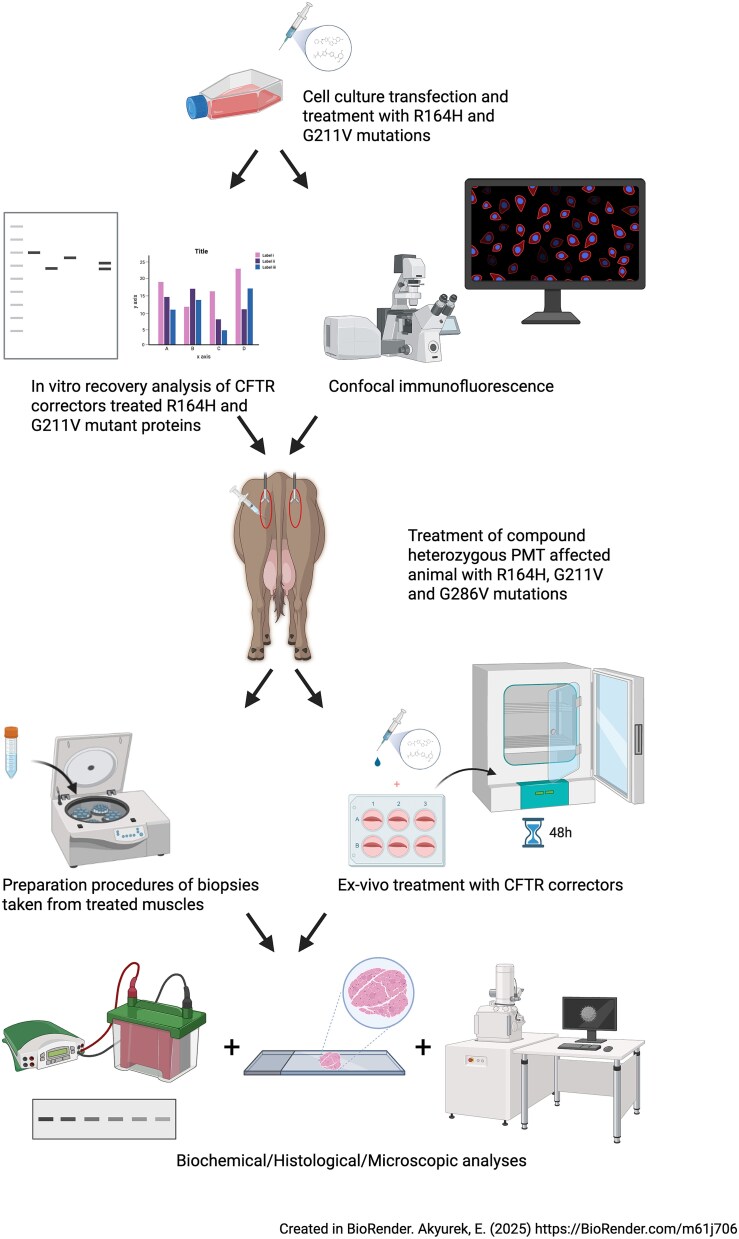

## Introduction

Brody disease is an ultra-rare autosomal recessive inherited disorder that impairs skeletal muscle function in humans [1]. The main clinical signs are exercise-induced muscular stiffness and delayed relaxation, which occur after even mild physical activity such as light running, climbing stairs, or playing piano [2]. The majority of Brody patients experience a marked increase in symptoms when exposed to cold [3]. The involved muscles are the voluntary muscles especially those used for the movement of legs and arms, but also the eyelids. Brody myopathy is caused by deficiency of the Sarco/Endoplasmic reticulum Ca^2+^-ATPase, isoform1 (SERCA1), arising from either missense and non-sense variants or in-frame deletions affecting the *ATP2A1* gene [4, 5].

In vertebrates, three genes (*ATP2A1–3*) encode the main SERCA protein isoforms 1, 2, 3 expressed in a tissue-depending fashion and according to the stage of development [6]. SERCA1 is the adult isoform exclusively expressed in fast-twitch type II skeletal muscle fibers. In muscle fibers the SERCA1 is a key participant in Ca^2+^ homeostasis, transporting the ion from cytosol back into the lumen of the sarcoplasmic reticulum (SR) and playing a crucial role in muscle relaxation and in the maintenance of resting intracellular Ca^2+^ concentration.

Bovine ‘congenital pseudomyotonia’ (PMT) is a very rare skeletal muscle disorder, clinically characterized by stiffness and delayed muscle relaxation, described first in Chianina and subsequently in Romagnola breeds [7, 8]. The clinical signs primarily manifest as muscle contractions triggered by physical activity, limiting the animals to a leisurely walking pace. When prompted to move faster, the muscles swiftly become rigid, causing a temporary loss of coordination and a ‘frozen’ gait. The hindlegs are more prominently affected, but the front legs also display signs of this condition. If the animals engage in prolonged physical activity, the stiffness leads to a temporary loss of movement, resulting in the animals falling to the ground. After a few seconds, the muscles relaxed, and the animals can resume normal movement. Animals also experience generalized muscle stiffening when startled or after transport stress.

As Brody myopathy, bovine PMT is an autosomal recessive inherited disorder caused by missense variants in the *atp2a1* gene. Three different causative missense variants in *atp2a1* have been found. The first reported was the p.Arg164His affecting Chianina and Romagnola cattle [9]. In addition, it was found that the variants p.Gly211Val and p.Gly286Val, are also responsible for PMT in Romagnola cattle [8]. All Chianina PMT affected animals result to be homozygous, while most Romagnola PMT affected calves are compound heterozygous, carrying two missense variants leading to Gly211Val (G211V) and Gly286Val (G286V) substitutions in one allele, and the Arg164His (R164H) mutation in the other allele. Recently, we demonstrated that in Romagnola breed the G286V variant is benign while the G211V is the pathogenic variant [10]. Irrespective of variant in *atp2a1* gene, a heterogeneous but selective reduction of expression of SERCA1 mutant protein was observed in SR membranes isolated from muscles of all PMT-affected cows. As a result, greater is the reduction, more severe is the disease. On the basis of clinical [7], genetic [9] and biochemical analyses [10, 11], our group identified bovine PMT as the real counterpart of human Brody disease.

Using *in vitro* and *ex vivo* experimental approaches involving heterologous cellular model overexpressing R164H and G211V SERCA1 mutants, or adult muscle fibers from a Chianina PMT affected subject respectively, we clarified the pathogenesis of bovine PMT [10, 12]. We demonstrated that both mutations generate proteins corrupted in proper folding that although catalytically active, were ubiquitinated and prematurely degraded by the ubiquitin-proteasome system. Interestingly, bovine PMT shares with Cystic Fibrosis (CF) the same pathogenetic mechanism [13, 14] of type II mutants of the Cystic Fibrosis Transmembrane Regulator (CFTR), which give rise to folding-defective proteins, affecting trafficking. This class of mutants, that includes the deletion F508 (F508del-CFTR) carried by 80–85% of the worldwide CF population, is recognized by the endoplasmic reticulum (ER) quality control system, tagged with ubiquitin and ultimately degraded by the proteasome.

In the last decade, several small molecules have been developed to treat CF [15]. Some of them are known for their ability to repair folding defective type II CFTR mutants and to recover them at the cell surface, which is why these molecules are named ‘correctors’ of CFTR protein.

At present, neither specific therapy [16] nor mouse model [17] exists for Brody myopathy. In this study we show that CFTR correctors are effective in recovering SERCA1 mutants causing bovine PMT. We report the effects of CFTR correctors, mainly the compound named C17, in both heterologous cellular systems, previously successfully used for investigating the pathogenetic mechanism of bovine PMT, and in SR membrane fractions isolated from muscle biopsies obtained from PMT affected cows. This animal model, despite being unconventional, is currently the unique mammalian model of human Brody myopathy. On the base of *in vitro* experiments and *in vivo* treatments, in this paper we propose a potential innovative pharmacological approach addressing a specific population of Brody patients in which the functionality in the mutated SERCA pump is maintained.

## Results

### Recovery *in vitro* of R164H and G211V SERCA1 mutants by means of CFTR correctors

It was established that by blocking the proteasome system with the well-known inhibitor MG132, both R164H and G211V SERCA1 mutant proteins causing PMT in bovine species, accumulated in heterologous cells lines at levels comparable to those of wild type (WT) SERCA1 [10, 12].

**Figure 1:**
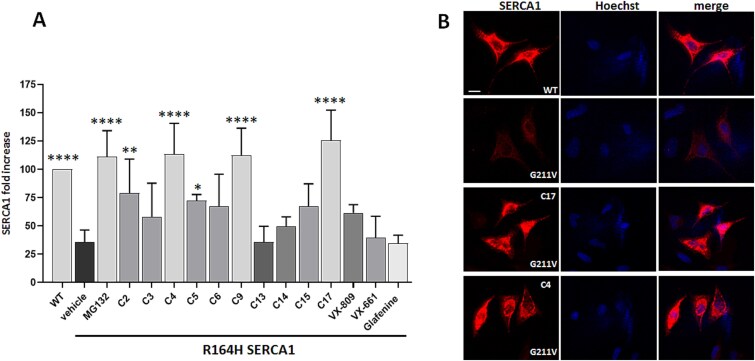
CFTR correctors promoted the rescue of R164H and G211V mutant SERCA1 in cells. (A) HEK293 cells transfected with WT and R164H mutated SERCA1 cDNAs were treated with the proteasome inhibitor MG132 (10 μM), the vehicle DMSO (0.1%), CFTR correctors C2 (5 μM), C3 (10 μM), C4 (10 μM), C5 (15 μM), C6 (10 μM), C9 (10 μM), C13 (5 μM), C14 (5 μM), C15 (15 μM), C17 (2 μM), VX-809 (10 μM), VX-661 (10 μM) and the molecule glafenine (10 μM). All molecules were dissolved in the DMSO. An equal quantity of protein from total cell lysates was separated by SDS-PAGE and subjected to immunoblot analysis with antibodies specific to SERCA1 and 43 kDa beta actin, used as loading control. The graph shows the average values (± SD) of SERCA1 expression determined by densitometric analyses of four independent experiments (n = 4). Values are expressed as percentage of the SERCA protein content present in cells expressing the wild type form. Statistical analysis was performed by one-way ANOVA test, multiple comparisons Dunnett test; ^****^P ≤ 0.0001; ^**^P ≤ 0.01; ^*^P ≤ 0.05. (B) HeLa cells were transfected with WT or with G211V mutated SERCA1 cDNAs, as indicated. Twenty-four hours after transfection, C17 (5 μM) or C4 (10 μM) CFTR correctors dissolved in DMSO (0.1%), were added. At the end of incubation time, cells were fixed and immunolabelled with monoclonal antibodies to SERCA1 and then incubated with the Alexa 568 fluorescence secondary antibody. Images were recorded at the same setting conditions and magnification. Scale bar 25 μm.

Here, heterologous cells were used to assess the response *in vitro* to twelve pharmacological molecules named CFTR correctors, due to their ability to rescue CFTR type II mutants causing CF in humans [15, 18]. These molecules are listed in Material and Methods and in Reference (19). HEK293 cells transfected with cDNA encoding R164H mutant SERCA1 protein, were incubated for 24 h with the CFTR correctors and glafenine at the concentrations reported in the legend of Fig. 1. The anti-inflammatory molecule glafenine, although being not a corrector, was found to increase expression of F508del-CFTR [20]. Cells transfected with the WT SERCA1 were used for comparison, while the proteasome inhibitor MG132 was utilized as positive control of rescue.

At the end of incubation period, total cell lysates were analyzed by western blot with specific antibodies to SERCA1 (Supplemental Fig. S1) and the protein expression was quantified by densitometric analysis (Fig. 1A). Values were reported as percentage of the SERCA protein content present in cells expressing the WT form. Results confirmed that the expression level of R164H SERCA1 mutant was lower than that of WT protein but, after treatment with MG132, a significant increase in content of the R164H mutant was detected (Fig. 1A). Results also showed that the incubation with CFTR correctors C17, C4 and C9, induced an increase in the expression level of SERCA1 mutant almost equivalent to that of MG132-treated cells (Fig. 1A). Other tested CFTR correctors including VX809 [21] VX661 [22] and the molecule glafenine, were less effective or almost totally ineffective in rescuing R164H mutant SERCA1.

Cells transfected with cDNA encoding WT or G211V SERCA1 mutant were immunodecorated with anti SERCA1 antibodies. Results showed that the expression level of G211V variant was qualitatively equivalent but remarkably lower than WT [10]. After incubation for 24 h with C17 or C4 correctors, the immunofluorescence pattern of G211V variant was increased to level similar to WT control (Fig. 1B).

### Recovery of mutant SERCA1 proteins *in vivo* after incubation with C17 CFTR corrector

The efficacy of C17 corrector to improve expression and trafficking of defective SERCA1 was validate *in vivo.* Experiments were carried out in two PMT affected calves from Romagnola breed, named case 1 and case 2 and admitted to Veterinary hospital seven months apart. Both affected subjects were compound heterozygous, carrying SERCA1 R164H mutation (found in the Chianina breed) [9] in one allele and missense variants G211V and G286V (the second one found to be benign) in the other allele [10]. On clinical examination at the time of admission, both patients were apparently healthy and walked normally at slow pace, without signs of muscle contractures. Muscle tone, tropism, growth, were all within the normal range and there was no evidence of percussion myotonia. Importantly, no abnormalities were observed in mental status, behavior, posture, balance, voluntary movement, or reflexes, whether they were related to peripheral or cranial nerves. Both cases showed exercised induced generalized muscle stiffness episodes.

**Figure 2:**
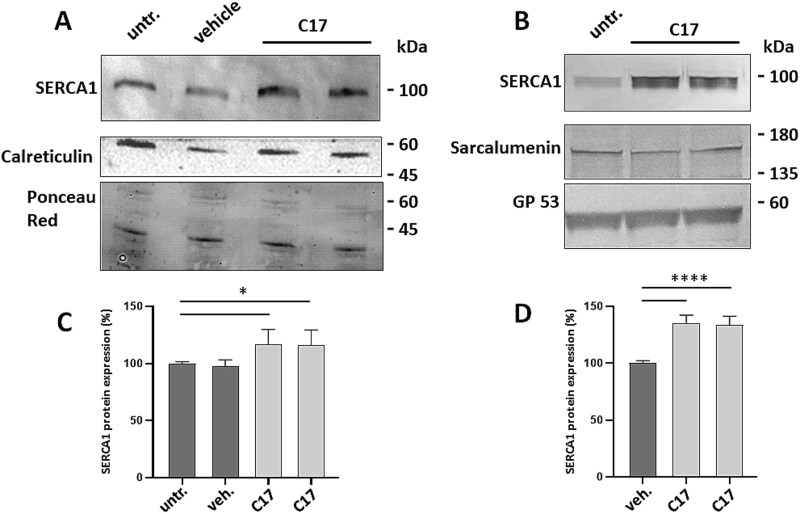
Expression level of mutated SERCA1 in microsomes isolated from skeletal muscle after treatment with corrector C17. Microsomal fractions enriched in content of SR membranes were obtained from semimembranosus muscle biopsies of case 1 and case 2 PMT affected calves. Biopsies were taken from two injection sites treated with C17, the contralateral treated with the vehicle or untreated sites for case 1 (A) or from two injection sites treated with C17 and the contralateral untreated site, for case 2 (B). Microsomal fractions were separated by SDS-PAGE and blotted onto nitrocellulose. The blots were incubated with antibodies to SERCA1 (protein loading was 3 μg/lane and 5 μg/lane in *a* and *B*, respectively). Staining with Ponceau red or incubation with the indicated antibodies (calreticulin and the non-junctional sarcalumenin and its splice variant 53 kDa glycoprotein), were used as loading control. A representative western blot is shown. Quantification by densitometric analysis of western blots from case 1 (C) and case 2 (D). The average amount of SERCA1 is expressed as fold increase (± SD) of the protein content compared with the contralateral untreated site. Data are from four technical replicates (western blots). Statistical analysis was performed by one-way ANOVA test—Multiple comparisons Dunnett test; ^****^P ≤ 0.0001; ^*^P ≤ 0.05.

PMT-affected patients were kept at rest to avoid contractual crisis and C17 CFTR corrector was injected intramuscularly in two different sites in the left semimembranosus muscle using a needle (Supplemental Fig. S2). The treatment was given every two days for a total of 3 and 5 injections for case 1 and case 2, respectively. Case 1 was treated with C17 with a dose of 50 μg/g of muscle, and case 2 with 100 μg/g of muscle. At the end of treatments muscle biopsies were taken from the two injection sites and from the contralateral sites, untreated or treated with vehicle. Biopsies were homogenized and sub-fractionated [11] to obtain a crude microsomal fraction enriched in content of SR membranes, a soluble supernatant and a myofibrillar fraction, while a small portion at sites of injections was employed to perform morphological analysis on thin sections. From case 1, a muscle biopsy was taken from the contralateral site treated with the vehicle used to emulsify the C17 molecule (Fig. 2A).

**Figure 3:**
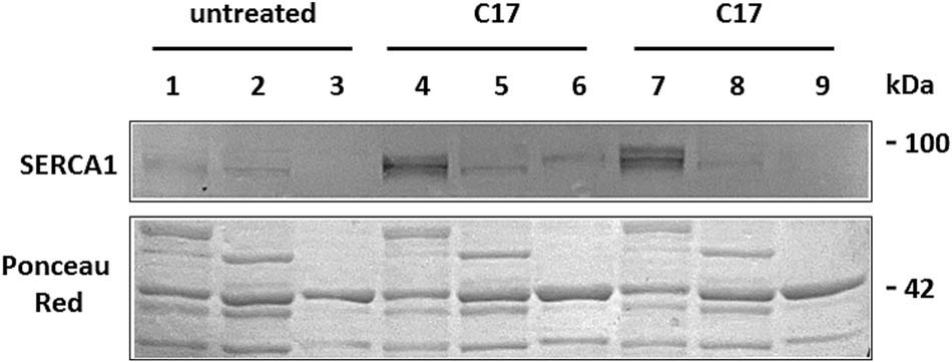
Distribution of SERCA1 protein within muscle subfractions. Muscle homogenates from the two injection sites treated with C17 and the contralateral untreated semimembranosus muscles of PMT-affected case 2 calf, were sub-fractionated as described in materials and methods to obtain a crude microsomal fraction enriched in content of SR membranes (lanes 1, 4, 7), a soluble supernatant (lanes 2, 5, 8), and a myofibrillar fraction (lanes 3, 6, 9). Proteins were separated by SDS-PAGE and blotted onto nitrocellulose. The blots were incubated with antibodies to SERCA1. The lower part of the panel shows Ponceau red staining, used as loading control.

Western blot analysis with antibodies to SERCA1 on SR microsomal fractions evidenced an increase of immunoreactivity in treated samples despite the heterogeneity of SERCA1 staining in PMT affected muscles (Fig. 2), which depends on the severity of the pathology as already described [8, 11]. The increase in SERCA1 mutant expression was more pronounced in case 2, in which the treatment was prolonged and C17 concentration enhanced. Rescued SERCA1 protein was detected exclusively in SR fractions, being totally absent from myofibrillar pellet and especially from soluble cytoplasm (Fig. 3).

**Figure 4:**
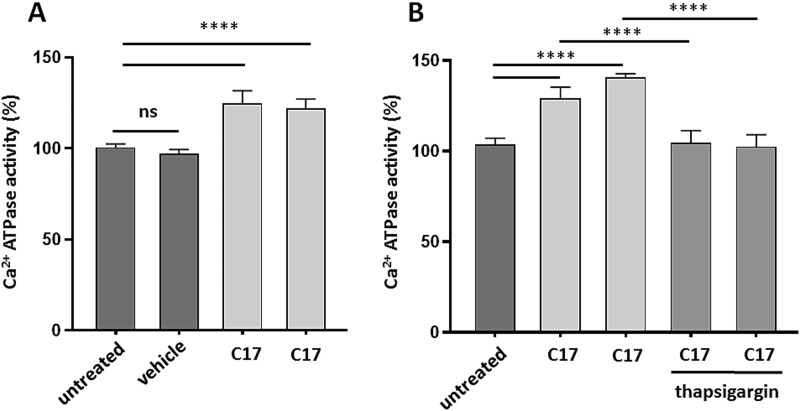
Ca^2+^-ATPase activity in SR microsomes isolated from semimembranosus skeletal muscle biopsies after treatment with corrector C17. Ca^2+^-ATPase activity assay at pCa5 was performed on SR microsomal fractions obtained from two injection sites treated with C17, the contralateral treated with the vehicle or untreated sites for case 1 (A), or from the two injection sites treated with C17 and the contralateral untreated site, for case 2 (B). Data (± SD) were obtained from four technical replicates in which each sample was tested in duplicate. Statistical analysis was performed by one-way ANOVA test—Multiple comparisons Dunnett test; ^****^P ≤ 0.0001; *^**^P ≤ 0.001.

The functional assay indicated that SR-enriched membrane fraction obtained from C17 treated muscle fibers displayed in parallel an increase in maximal Ca^2+^-ATPase activity, measured at saturating calcium concentration (pCa 5) (Fig. 4A and B) which was abolished by the use of thapsigargin, a well known inhibitor of SERCA pumps but not of other ATPase such as those of plasma membrane (Fig. 4B).

**Figure 5:**
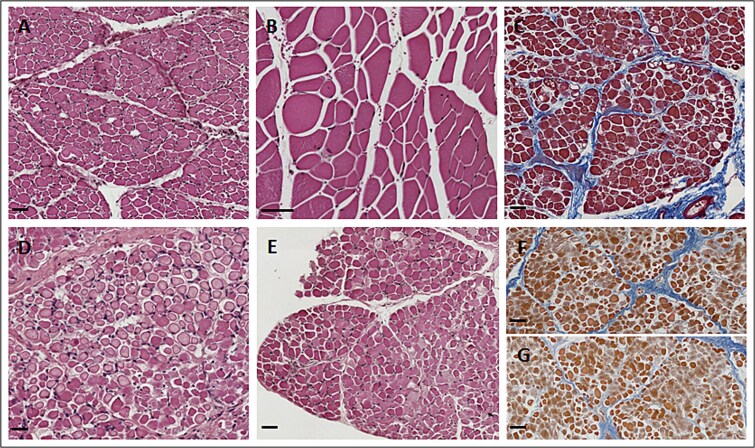
Histological staining on semimembranosus muscle sections from PMT-affected subjects after treatment with C17 CFTR corrector. Transversal sections from semimembranosus muscle biopsies taken at the end of muscle treatment protocol with C17 molecule. Biopsies were taken from the injection sites (A, B, C) and contralateral untreated site (G) for case 1; from injection sites (D, E) and contralateral untreated site (F) for case 2. Sections were stained with Hematoxylin and eosin (H&E) (A, B, D, E) or azan–Mallory (C, F, G) methods. Scale bars 50 μm.

Histopathological examination of case 1 muscle biopsy showed that the majority of fibers appeared normal in diameter while a few were enlarged and round shaped, likely closed to degeneration. Pale degenerated fibers were also found. In case 2, in which the pathology was more severe based on SERCA1 SR content, degenerative changes evidenced a variability in fibers diameter and enlarged round shaped fibers. No inflammatory cell infiltration was evidenced, but perimysial fibrosis was observed in samples from both case1 and case 2 PMT affected subjects (Fig. 5).

### Quantitative rescue of mutant SERCA1 proteins after incubation with C17 and C4 CFTR correctors in *ex vivo* experiments

**Figure 6:**
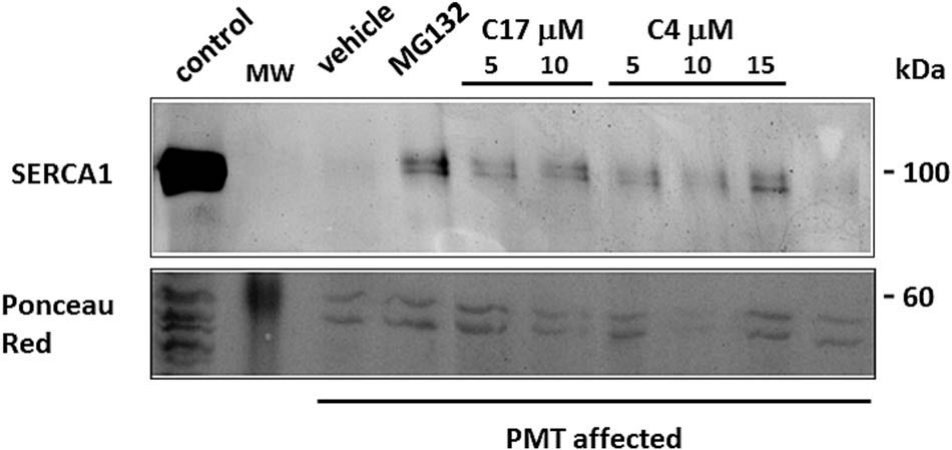
*Ex vivo* treatment with C17 CFTR corrector of semimembranosus muscle fibers from case 1 PMT affected calf. Bundles of fibers from muscle biopsy taken from contralateral untreated muscle of PMT-affected case 1, were maintained under tissue culture conditions (untreated) or were incubated with MG132 (5 μM), CFTR correctors C17 (5 and 10 μM) and C4 (5, 10 and 15 μM) at different concentrations or with their vehicle, DMSO (0,1%), as indicated. After treatment, fibers were homogenized and sub-fractionated as described in materials and methods. An equal quantity of proteins from the crude microsomal fraction enriched in content of SR was separated by SDS-PAGE and blotted into nitrocellulose (protein loading 3 μg/lane). The SR membrane fraction from healthy Romagnola calf was used as control. Blots were incubated with antibody to SERCA1. The lower panel is Ponceau red staining of the membrane fractions, used as loading control.

A small portion of muscle biopsy from contralateral untreated muscle biopsy of case 1 was used to isolate tiny bundles of adult muscle fibers. The bundles of fibers were maintained under tissue culture conditions as explants and were incubated with C17 or with C4 correctors at different concentrations. Incubation with the proteasome inhibitor MG132 was used as positive control. At the end of incubation period, SR membranes enriched fractions were isolated from these small explants. The immunoblot analysis with antibody to SERCA1 on SR fractions showed that the treatment with C17 or C4 correctors efficiently increased SERCA1 mutant expression, confirming *in vivo* experiments and being in close agreement with *in vitro* results on transfected cells (Fig. 6).

### Ultrastructural analysis of PMT- affected muscle after contractural crisis

**Figure 7:**
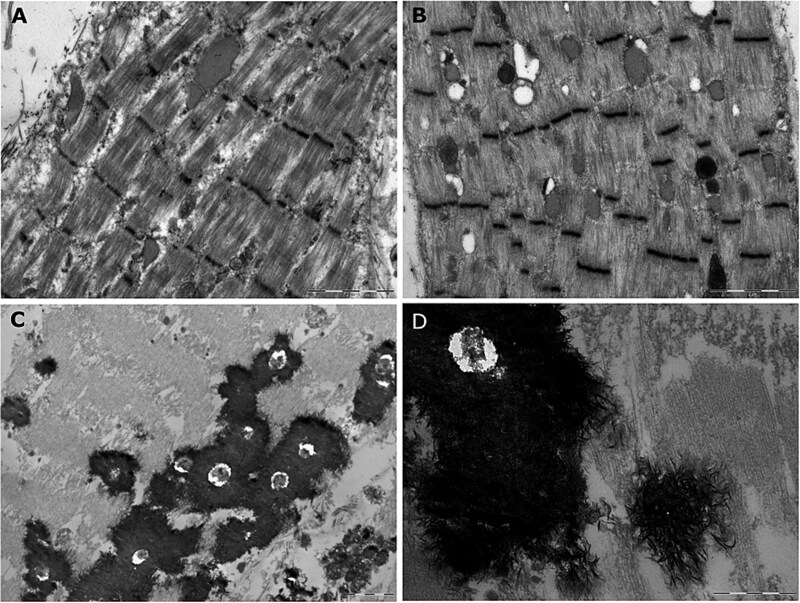
Ultrastructural studies of skeletal muscles from bovine PMT- affected case 1. Electron microscopy of semimembranosus (A) semitendinosus (B) and quadriceps (C, D) muscle biopsies from PMT-affected subject. Samples were collected after a severe contractural crisis. Scale bars, 2 μm (a, B, C) and 1 μm (D).

Two months after the end of treatment, case 1 subject walking on the uneven ground, accidentally experienced stiffness in his limbs. This event led to a loss of motor co-ordination and balance resulting in the falling to the ground of the subject. Despite many attempts the subject was unable to rise. Indeed, the repetitive and sustained contractions caused a marked and extensive rhabdomyolysis as suggested by haematological analysis showing a significant increase in serum creatine kinase (CK) (9974 U/l; reference 272 U/l). Due to severe emaciation and extensive muscle damage secondary to the severe muscle contractions, the subject was euthanized. Immediately after euthanasia muscle sample from three different muscles were collected and used for histological examination (Supplemental Fig. S3) and ultrastructural studies (Fig. 7). Histological examination showed endomysial basophilic inflammatory cells infiltration and hyper contracted fibers with fragmented sarcoplasm in Semimembranosus and Quadriceps muscles, respectively. Ultrastructural studies showed that in Semimembranosus muscle the myofibrillar architectural pattern appeared maintained but loss of filaments was focally evident (Fig. 7A). Areas of myofibrillar disarray exhibiting loss of normal cross-striation, were evident. Dilated SR cisternae corresponding to Z-lines and containing a medium electron-dense matrix with amorphous fibrillar structure, were also visible. Few isolated phagolysosomal vacuoles were detectable below the sarcolemma. The submicroscopical organization of the Semitendinosus (Fig. 7B) muscle closely resemble that of Semimembranosus. The dilated SR cisternae seemed to be smaller and numerous and few phagolysosomal vacuoles were also present. By contrast, in Quadriceps muscle the myofibrillar pattern was completely destroyed with loss of cross striation and presence of aggregates of filaments (Fig. 7C). Many electrondense inclusions are clearly visible constituted by an accumulation of crystals likely representing Ca^2+^ aggregates (Fig. 7C and D).

### C17 cytotoxicity in bovine and human cell lines

**Figure 8:**
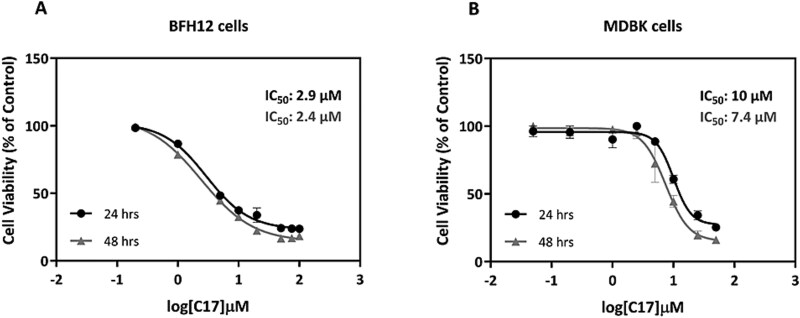
Cell viability assays in bovine cell lines treated with C17 corrector at different concentrations. Dose–response curves of C17 in BFH12 (A) and MDBK (B) cells after 24 and 48 hrs of incubation. Data are expressed as the mean viability rate (%) (± SD) of three biological replicates (n = 3 independent experiments), each performed in sextuplicate.

Owing to ethical issues about the execution of *in vivo* preclinical trials in the bovine species, the potential adverse effects of C17 corrector were tested on cultured cells from bovine liver and kidney, which represent two hinge tissues involved in pharmacokinetics. Specifically, fetal bovine hepatocyte-derived (BFH12) [23] and Madin-Darby Bovine Kidney (MDBK) [24] cell lines were used. Cells were exposed to eight increasing concentrations of C17 for 24 and 48 h; then, dose–response curves were built to define the corresponding TC50 values (Fig. 8). After 24 h of incubation, the estimated TC50 for BFH12 and MDBK cells was 2.9 μM (R^2^ = 0.98) and 10 μM (R^2^ = 0.96), respectively (Fig. 8A and B). At 48 h of incubation the TC50 only slightly decreased in both *in vitro* models, showing values of 2.4 μM (R^2^ = 0.99) and 7.4 μM (R^2^ = 0.94) in BFH12 and MDBK cells, respectively (Fig. 8A and B). Notably, the highest concentrations tested (*i.e.* 50 and 100 μM) never caused 100% of cell death in both bovine cell lines.

For comparative purposes, we evaluated C17 cytotoxicity also in a human hepatic cell line, *i.e.* the HepG2 cell line (Supplementary Fig. S4). At 24 h of incubation, C17 did not impair the viability of HepG2 cells (estimated TC50 > 100 μM), and at 48 h, the TC50 was ~ 5 times higher (13.4 μM, R^2^ = 0.94) than in BFH12 cells (2.4 μM: Fig. 8B), suggesting a lower cytotoxicity of C17 in the human liver cell line compared to bovine foetal hepatocytes.

## Discussion

Rare diseases, known as conditions affecting a small number of individuals compared to the general population [25], are further classified as rare or ultra-rare [26]. About 80% are of genetic origin [27] and many are skeletal muscle disorders characterized by abnormalities on voluntary muscle fibers, limiting daily living and often resulting in progressive reduction in muscle functions. For most rare and nearly all ultra-rare pathologies, appropriate pharmacological treatments are not currently available so that they are commonly defined ‘orphan diseases’. This is the case of Brody myopathy, an ultra-rare genetic disorder characterized by muscle stiffness and impairment of relaxation. Brody patients are at present treated with generic muscle relaxant drugs that prevent Ca^2+^ release from SR [16, 28]. Myorelaxant drugs alleviate signs and symptoms but do not act on the pathological mechanism of the disease. In addition, they are unsuitable for long-term treatments due to severe side effects [29].

Bovine PMT is the true animal counterpart of human Brody myopathy [7, 11]. Its pathogenetic mechanism has been clarified [10, 12]. The SERCA1 variants generate folding-defective proteins that despite retaining their catalytic activity, are promptly disposed of by the ubiquitin-proteasome system, resulting in a 'loss of function'. In fact, the consequent reduction of SERCA1 at SR membranes of muscle fibers, is responsible for impairment of muscle relaxation after contraction. As in Brody myopathy, the prolonged high concentration of Ca^2+^ in intracellular space of skeletal muscle fibers arises from the difficulty in removing the ion due to the SERCA1 protein deficiency [2, 11]. Indeed, *in vivo* and *ex vivo* experimental approaches showed that SERCA1 mutants, rescued by proteasomal inhibition, are fully capable to maintain cytoplasmic Ca^2+^ at similar levels to those observed in cells expressing the WT protein [10, 12].

The knowledge of PMT pathogenesis, allowed us to propose a potential pharmacological approach to cure Brody myopathy, based on the repositioning strategy of ‘CFTR correctors’. These small molecules are known for the ability to promote the recovery and trafficking of folding-defective class II mutants of CFTR (*e.g*. F508del-CFTR), causing CF. The retention of functionality in the mutated SERCA1 pump, represents the fundamental requisite for the therapeutic effectiveness of the pharmacological strategy here proposed.

In this study, using *in vitro, in vivo* and *ex vivo* experimental approaches, we investigated CFTR correctors specifically C17 molecule, as potential therapeutic candidate for Brody myopathy.

Using as *in vitro* model heterologous cell lines overexpressing R164H SERCA1 mutant, we confirmed our hypothesis, as some CFTR correctors successfully rescued this mutant. Corrector C17 in particular (Fig. 1A and Supplemental Fig. S5), but also correctors C4 and C9 were the most effective. *In vitro* treatment with C17 and C4 correctors had beneficial effect also in G211V SERCA1 mutation. The molecules VX809 and VX661, already approved by FDA for the treatment of CF in combination with other class of molecules (Orkambi and Trikafta respectively) [22], were not highly efficient in rescuing SERCA1 mutant. This is not surprising considering the high specificity of VX809 for CFTR protein [21] and the shared mechanism through which VX809 and VX661 are supposed to selectively bind to defective CFTR channel.

The compounds C17 and C4 which belong to the same chemical family (being both bithiazole derivatives), have already proven to be effective in rescuing expression of mutant proteins whose final destination is the plasma membrane, as shown *in vitro* for ATP8B1 mutant [30]. The ability of these two molecules in recovering expression and trafficking of proteins to plasma membrane from skeletal muscle fibers (sarcolemma) has also been demonstrated *in vitro* for several folding defective α-sarcoglycan [19, 31] and β-sarcoglycan [32] (SG). Mutated α-SG and β-SG proteins cause limb-girdle muscular dystrophy R3 (LGMDR3) and R4 (LGMDR4) respectively, severe muscular dystrophies belonging to the large family of orphan diseases. Recently, the *in vivo* validation of corrector C17 as potential therapeutic compound for LGMDR3 has been proved by Scano et al. [33]. For these reasons, we mainly focused our attention on C17 that became our lead compound and was used for *in vivo* validation as potential therapeutic molecule to rescue defective SERCA1 proteins causing Brody myopathy.

Usually, *in vivo* preclinical studies are performed in mouse, being the most popular animal model of human diseases in drug development research. However, sometimes this model, failing to mirror the disease phenotype, results unsuitable to study certain human pathologies.

It is well known that in mouse, skeletal muscle physiology is strongly dependent on fast-twitch muscle activity [34]. Wide differences in fibers type composition of diaphragm muscle has been described between mice and large mammals, including humans [17, 35, 36]. Fast-twitch fibers account for more than 90% of diaphragm in mice compared to about 60% in humans. A SERCA1 knock-out mouse was generated by Pan et al, [17]. Newborn mice showed limb contractures as in Brody patients, but also gasping respiration and severe diaphragm dysfunction (never described in Brody patients). As a consequence, mice died by respiratory failure within two hours of birth.

To overcome the lack of mouse model for Brody myopathy, the strategy we adopted to validate *in vivo* C17 corrector was to perform studies using the unique recognized, albeit unconventional, mammal model, *i.e*. the cattle PMT. Being large animals not suitable for systemic administration in drug development research, we treated locally PMT-affected cattle. The corrector C17 was injected on the outer layer of the semimembranosus skeletal muscle, a representative fast-twitch muscle in cattle based on the MHC isoforms composition [11]. The procedure was possible since this muscle is superficially located on the pelvic limb and so easily accessible by needle placement. The molecule was administered to two compound heterozygous Romagnola breed PMT affected cows, carrying the mutation R164H in one allele and the substitution G211V in the other allele. We were unable to quantify the degree of expression of each allele in the treated animals. However, the treatment of heterozygous patients provided the opportunity to simultaneously evaluate the efficacy *in vivo* of the C17 corrector on the two distinct SERCA1 variants. The *in vitro* data suggested that both mutations are amenable to rescue, supporting the assumption that a similar outcome occurs *in vivo*.

It has been already demonstrated that PMT affected subjects were heterogeneous with respect to the SERCA1 muscle content [8, 11], being the degree of reduction of protein expression from mild to severe. Here we show that the local administration of C17 corrector resulted in the rescue of R164H/G211V mutant SERCA1 both in case 1 and in case 2, where the pathology is more severe as evidenced by the faintly detected SERCA1 protein band before treatment and by more manifest muscle damage. The SERCA1 protein accumulates exclusively in SR fraction vesicles which exhibited an increase in thapsigargin sensitive Ca^2+^-ATPase activity, confirming that the rescued SERCA1 Ca^2+^-ATPase is potentially capable to maintain Ca^2+^ homeostasis.

We have previously shown by ultrastructural studies that if animals were kept at rest, the general structure of muscle fibers was normal and triads were regular in size and distribution [12]. While walking on uneven ground of the straw-manure bedding, case 1 accidentally experienced limbs contracture crisis that caused the animal to fall to the ground. This event happened two months after the end of the treatment, in the coldest days of January. As for Brody’s patients, the increase of PMT symptoms was observed in cows when exposed to low temperatures. This condition together with the numerous attempts to rise from the ground, caused a worsening in the contracture crisis. The progression of the severity of contracture was easily observed by irregularities in myofibrillar organization and ultrastructural abnormalities, especially in Quadriceps, one of the critical muscles for normal rising in cattle. The large amount of intracellular Ca^2+^ ions accumulated during repetitive attempts to get up and the inability to pump them back into SR due to the reduced amount of Ca^2+^-ATPase are probably the cause of deposition of large crystal structures found in this muscle. Calcium deposits contribute to disruption of myofibrillar arrangement and consequently loss of force, creating a vicious circle that leads to a progressive muscle degeneration.

Overall, our results clearly demonstrated that the C17 corrector has beneficial effects on SERCA1 mutants causing PMT. Thus, this potential therapeutic approach could be used in the treatment of that specific population of Brody patients in which a reduction in expression but not in activity of the pump has been documented. In fact, only functionally active SERCA1 when located at the SR membrane, could restore the efficient control of Ca^2+^ homeostasis and prevent the appearance of signs and symptoms of the pathology. Moreover, ultrastructural results suggested that the therapy should start at early stage, *i.e.* before Ca^2+^ overload resulted in muscle damages and contributed to muscle fibers degeneration.

The drug dose used to treat PMT case 1 was based on the daily recommended dose of Orkambi (approximatively 11–16 μg/g depending on average weight of an adult). No behavioral differences or signs of distress, but mostly no adverse local reactions were noted, even in PMT case 2 where the dose was doubled and the treatment prolonged. Even after chronic systemic treatment with C17 (intraperitoneal injection up to 5 weeks) in LGMDR3 model mice with humanized hind limbs, no evident sign of toxicity on liver and kidney was observed at the macro and microscopic level, as well as no effect on growth and behavior of mice [33]. In a recent work, we have treated the zebrafish line accordion with C17 corrector [37]. This line is characterized by a point mutation in the *atp2a1* gene that replaces serine with phenylalanine at position 766 (S766F) of SERCA1. This mutation does not preserve functional properties of the pump, which are essential for the efficacy of the potential pharmacological therapy here proposed. As a result, treatment with C17 did not led a substantial recovery of locomotor activity or morphological traits in this zebrafish model. However, our results indicate that C17 CFTR corrector (at concentration used in our study) has no toxic effects and is well tolerated also in zebrafish.

Although the clinical observations reported above were encouraging and indicative of a scant toxicity of C17 in both bovine and murine animal species following local and systemic treatments, we decided to screen the potential adverse effects of C17 in bovine species-specific *in vitro* models deriving from two organs playing a pivotal role in drug metabolism, namely liver and kidney. These additional analyses were performed because large animals are not commonly used in kinetics studies of new candidate drugs, being, as mentioned above, not properly suitable for systemic administration. The results here obtained show that C17 is not highly toxic in bovine cells, since the highest concentrations used (*i.e.* 50 and 100 μM) never lead to 100% cell death in both cell models. In addition, in MDBK cells a TC50 between 7 and 10 μM was calculated. Worth noting, compounds with EC50 and Cmax between 10 nM and 10 μM are usually considered safe drugs, because Cmax > 10 μM, required for efficacy at the target site, may cause off-target activation and pharmacokinetic toxicity [38]. Consequently, we do not retain that these concentrations could represent a concrete risk of untoward/toxic effects. Nevertheless, to draw definite conclusions on the toxicity of the compound for a potential clinical development, the therapeutic index (TC50/EC50) and Cmax at the target site should be calculated; these PK-PD analyses are ongoing. To further corroborate our assumption, we tested the same concentrations of C17 in a hepatic cell line of human origin (HepG2), again demonstrating a low toxicity of the molecule as the TC50 was > 100 μM at 24 h and 13.4 μM at 48 h of incubation (Supplementary Fig. S4). Presumably, the higher sensitivity observed in BFH12 cells may be attributed to the fetal origin of the *in vitro* model; indeed, a differential expression of cytochrome P450 3A isoforms (the foremost family of xenobiotic metabolizing enzymes in humans) [39] between BFH12 cells and adult bovine liver tissue has been previously described [40].

The main limit of these *in vivo* studies is sample size. Bovine PMT, as Brody disease, is a very rare muscle myopathy and Chianina cattle is the most famous Italian breed for meat quality (Fiorentina steak). The *ATP2A1* genetic test introduced around 2010, the easy detection of PMT carriers and the subsequent selection against SERCA1 mutations, have enabled cattle breeders to implement these notions in their breeding strategy. As a result, the number of individuals affected by this genetic disease is drastically reduced [41].

In conclusion, in this paper we provide for the first time, direct evidence that CFTR correctors, particularly C17, successfully rescue SERCA1 mutants both *in vitro* and notably *in vivo* models.

Our aim now is to translate into therapy the use of the C17 CFTR corrector molecule to cure defective but functional SERCA1 proteins causing Brody myopathy. Moreover, we are also aimed in extending our therapeutic approach to other orphan diseases, sharing with CF, Brody myopathy and LGMDR3 a similar pathogenic mechanism.

## Materials and methods

### Chemicals and reagents

Glafenine and MG132 were from Sigma-Aldric; VX809 and VX770 were from Selleck Chemicals; C2, C3, C4, C9, C17 were a kind gift of the Cystic Fibrosis Foundation; C5, C6, C13, C14 and C15 were from Exclusive Chemistry; C4 and C17 were from Rosstek Limited (Paralimni, Cyprus). All compounds were dissolved in dimethyl sulfoxide and the working solution prepared to have the same content of vehicle (0.1%) in each treatment.

Dulbecco’s modified Eagle’s high glucose medium (DMEM), dimethyl sulfoxide (DMSO), dexamethasone, L-alanyl-L-glutamine and insulin from bovine pancreas were from Sigma-Aldrich (St. Louis, MO, USA). Williams’ Medium E, penicillin/streptomycin, fetal bovine serum (FBS), MEM-EARLES (EMEM) medium, non-essential amino acids were acquired from Life Technologies (Carlsbad, CA, USA).

### Plasmids, cell culture, transfection and treatment

The full-length cDNA encoding adult rabbit or bovine wild-type (WT) SERCA1 cloned in the pcDNA3 mammalian expression vector. The R164H and G211V substitutions were generated on SERCA1 cDNA of rabbit or bovine origin respectively, as previously described [10, 12].

HEK293 and HeLa cells (ATCC, Manassas, VA, USA) were grown in DMEM supplemented with 10% FBS. HEK293 cells were transfected the day after with wild-type (WT) or R164H SERCA1 mutant cDNA, using TransIT-293 (Mirus Bio) transfection reagents, according to the manufacturer's instructions. Twenty-four hours after transfection, medium was replaced with DMEM supplemented with 2% FBS containing CFTR correctors and glafenine dissolved in DMSO at concentration indicated in figure legends, or with DMSO alone (final concentration 0.1%). After treatment, HEK293 cells were washed twice with ice cold phosphate-buffered saline (PBS) and lysed with 5% sodium deoxycholate supplemented with protease inhibitors (Sigma-Aldrich, St. Louis, MO, USA). HeLa cells were transfected with WT and G211V SERCA1 mutant cDNA using jetOPTIMUS® DNA (Polyplus Transfection, New York, NY, USA) transfection reagents, according to the manufacturer’s instructions.

### Confocal immunofluorescence

At the end of treatment with CFTR correctors, HeLa cells were gently washed with PBS pH 7.4 and fixed with 4% paraformaldehyde for 15 min at room temperature. Cells were permeabilized in 0.5% Triton X-100 in PBS and immunodecorated with primary antibody, mouse monoclonal against SERCA1 (clone VE121G9, dilution 1:500, ThermoFisher Scientific, Waltham, MA, USA) and subsequently with Alexa Fluor 568 red secondary antibody as described [35]. Nuclei were stained with Hoechst 33342 (dilution 1 μg/ml, ThermoFisher Scientific, Waltham, MA, USA). Confocal microscopy was performed using a TCS-SP5 II confocal laser scanning microscope (Wetzlar, Germany).

### Clinical investigation, DNA samples and genotyping

An eleven-month-old Romagnola young bull (case 1) weighting 80 kg and a one-month-old Romagnola male calf (case 2) weighting 40 kg were referred to the Clinic for Ruminants of University of Bologna due to suspicion of bovine PMT.

### DNA samples and genotyping

Genomic DNA was isolated from EDTA-venous blood of affected animals collected at the time of admission to the clinic. DNA was extracted using the Promega Maxwell RSC DNA System (Promega, Dübendorf, Switzerland). Variants ATP2A1:p.Arg164His, ATP2A1:p.Glu211Val and ATP2A1:p.Gly286Val were genotyped by Sanger sequencing of PCR products as described before [8, 9].

### Pharmacological treatment

Experimental procedures were carried out in accordance with the recommendations of the European Legislation for the Protection of Animals used for Scientific Purposes (Directive 2010/63/EU) and of the Italian Parliament (L.LGS n26/2014). Pharmacological treatments and biopsies were authorized by the Italian Ministry of Health, ID 1251/2021.

For the pharmacological treatment, CFTR corrector C17 at the dose of 50 μg/g of muscle (case 1) and 100 μg/g of muscle (case 2) was used. C17 was dissolved in 5% DMSO, 5% Kolliphor-EL (Sigma-Aldrich) in physiological solution.

The region of the semimembranosus and semitendinosus muscles underwent trichotomy before beginning the pharmacological treatment. A mold was made to draw the bridges and the exact dimensions where the treatment injections would have to be given. The sites of treatment were marked with a permanent marker to allow to perform the injection always in the same point of injection. The treatment was applied intramuscularly in two different sites in the left semimembranosus muscle using a 25G (0.5 × 25 mm) needle. The treatment was given every two days for a total of 3 days of treatment for case 1 and 5 days of treatment for case 2.

### Muscle biopsy

Muscle biopsies were performed in lateral recumbency. Sedation with xylazine 0.05 mg/kg IM (Rompun, Elanco, Italy) was given. The surgical site was prepared for aseptic surgery. A 1 × 1 cm square of semimembranosus muscle biopsy was obtained from the two treatment injection sites and the contralateral untreated semimembranosus muscle in both animals 24 h after the last treatment injection. The skin was closed with simple interrupted sutures with 1–0 monofilament nylon. Muscle samples were fixed in liquid nitrogen at −200°C and then stored at −80°C. Ketoprofen (3 mg/kg; Zooketo, Elanco, Italy) was given before the biopsy and for two days after the biopsy.

### Preparative procedures and biochemical assay

Biopsies were homogenized as previously described [11] to obtain a crude microsomal fraction enriched in content of SR membranes, a final supernatant representing the soluble sarcoplasm and the myofibrillar fraction. Microsomal SR membrane fractions were resuspended in 0.3 M sucrose, 5 mM imidazole, pH 7.4 containing protease inhibitors and stored at −80°C.

ATPase activity of the SR microsomal fractions was measured by determination of NADH oxidation coupled to an ATP regenerating system adapted for use on a 96-well microplate, as previously described [10, 42]. In experiments with thapsigargin, the concentration used was 0.1 μM. The absorbance change at 340 nm was monitored using the EnVision (PerkinElmer) plate reader. Technical duplicates were performed for each experiment.

### *Ex-vivo* treatment with CFTR correctors

A small portion of freshly isolated muscle biopsy was divided into small bundles of fibers and placed in dishes as described [12]. Muscle bundles were incubated for 24 h with C4 and C17 CFTR correctors at different concentrations, with MG132 and with the vehicle (DMSO). The crude membrane fraction, was isolated by differential centrifugations [12].

### Gel electrophoresis and immunoblotting

Proteins were quantified by the Bicinchoninic Protein Assay Kit (Quantum Pro-tein Assay Kit, EuroClone Pero, MI, Italy). Proteins were resolved by SDS-PAGE and blotted onto nitrocellulose. The blots probed with mouse-monoclonal antibodies against SERCA1 (dilution 1:5000), beta-actin (clone AC-15, dilution 1:5000, Sigma Aldrich, St. Louis, MO, USA), sarcalumenin and its splice variant 53 kDa glycoprotein (clone XIIIC4, dilution 1:5000, ThermoFisher Scientific, Waltham, MA, USA) and rabbit-polyclonal to calreticulin (dilution 1:30.000, Sigma Aldrich, St. Louis, MO, USA). The membranes were then incubated with the appropriate secondary antibody. Densitometric scanning of protein bands was carried out using iBright 1500 (ThermoFischer Scientific, Waltham, MA, USA).

### Histological analyses

Muscle samples were fixed in 4% paraformaldehyde at 4°C, washed in phosphate-buffered saline and dehydrated through a graded series of ethanol. Samples embedded in paraffin were cut at 5 μm and stained with hematoxylin and eosin (H&E), or Azan–Mallory methods. Images were acquired by the automated slide scanner (Axioscan 7, Carl Zeiss Microscopy, GmbH, Jena, Germany).

### Transmission Electron microscopy analysis

Semimembranosus, Semitendinosus and Quadriceps muscle samples were collected from euthanized case 1. Samples were immediately fixed with 2.5% glutaraldehyde in 0.10 M cacodylate buffer and post-fixed with 1% OsO4 in the same buffer, dehydrated in ethanol, and embedded in epoxy resin. Ultrathin sections, obtained with an ultramicrotome, were counter stained with uranyl acetate and lead citrate and examined by a Philips CM100 transmission electron microscope (Philips, Amsterdam, The Netherlands).

### C17 cytotoxicity in bovine and human cell lines

The BFH12 (bovine SV40 large T-antigen-transduced fetal hepatocyte-derived) cell line was provided by Dr Axel Schoeniger (Institute of Biochemistry, University of Leipzig, Leipzig, Germany). Cells were maintained in 25 cm^2^ flasks (Sarstedt, Verona, Italy) and cultured in Williams’ E medium supplemented with 5% FBS, 1% penicillin/streptomycin, 2 mM L-alanyl-L-glutamine, 100 nM dexamethasone and 0.2 U/ml insulin.

The MDBK (Madin-Darby Bovine Kidney cell line, CCL-22™) cell line was purchased from ATCC and maintained in 25 cm^2^ flasks. Cells were cultured in EMEM medium supplemented with 10% FBS, 1% L-alanyl-L-glutamine, 1% penicillin/streptomycin and 1% non-essential amino acids. The HepG2 (human liver cancer cell line, HB-8065™) cell line was kindly provided by P. Dubreuil (INSERM U1068, CNRS, Marseille, France). Cells were grown in 75 cm^2^ flasks in EMEM medium containing 10% FBS, 1% L-alanyl-L-glutamine and 1% penicillin/streptomycin.

BFH12 cells were seeded in 96-well flat-bottom plates at a density of 6 × 10^3^ cells/well. Four days after seeding, cells were exposed to eight increasing C17 concentrations for 24 and 48 h. Conversely, MDBK and HepG2 cells were plated at a density of 4 × 10^4^ cells/well, and were exposed to C17 the day after seeding for the same time intervals. For BFH12 and HepG2 cells a concentration range comprised within 0.2 and 100 μM was selected, while for MDBK cells a different range was used (i.e. 0.05–50 μM). For C17 dissolution DMSO was used as the vehicle at the final concentration of 0.1%. Cells exposed to 0.1% DMSO only were used as control. The cell treatment was performed using media without FBS to avoid C17 precipitation.

C17 cytotoxicity was assessed by using the WST-1 Cell Proliferation Reagent assay kit (Roche, Basel, Switzerland), following manufacturers’ instructions. The cell viability was expressed as the percentage relative to that of cells exposed to the vehicle only (i.e. DMSO 0.1%). Three independent biological replicates (n = 3 independent cell culture experiments) per cell line were performed. Each concentration was tested in sextuplicate.

### Statistical analysis

Dose–response curves were built using GraphPad Prism software (version 8.0.2, San Diego, CA, USA). Viability rates of the treated cells compared to control cells (DMSO 0.1%) were reported, and a non-linear regression (log(inhibitor) vs. normalized response, variable slope) was built. The half-maximal inhibitory concentration (C50) and the goodness of fit (R^2^) were provided by the software.

## Supplementary Material

Supplemental_Data_HMG-2025-OA_00282_Aky_rek_ddaf142
