# Supplementary material for: CFTR corrector C17 rescues defective SERCA1 in bovine pseudomyotonia: a potential therapy for Brody myopathy
Source: Hum Mol Genet. 2025 Nov 6;34(24):2042–54. doi: 10.1093/hmg/ddaf142 (PMC12681263; doi:10.1093/hmg/ddaf142)

**Supplemental Figure S1 A and B**.

**CFTR correctors promoted the rescue of R164H mutant SERCA1 in HEK293 cells.** Cells transfected with WT and R164H mutated SERCA1 cDNAs were treated with the proteasome inhibitor MG132 (10 μM), the vehicle DMSO (0.1%), glafenine (10 μM) and CFTR correctors C2 (5 μM), C3 (10 μM), C4 (10 μM), C9 (10 μM), C17 (2 µM), VX-809 (10 μM), VX-661 (10 μM). All molecules were dissolved in the DMSO. Representative western blot of total protein lysates from HEK293 cells. Membranes were incubated with primary antibodies against SERCA1 and beta-actin, used as loading control. A representative Western blot is shown.


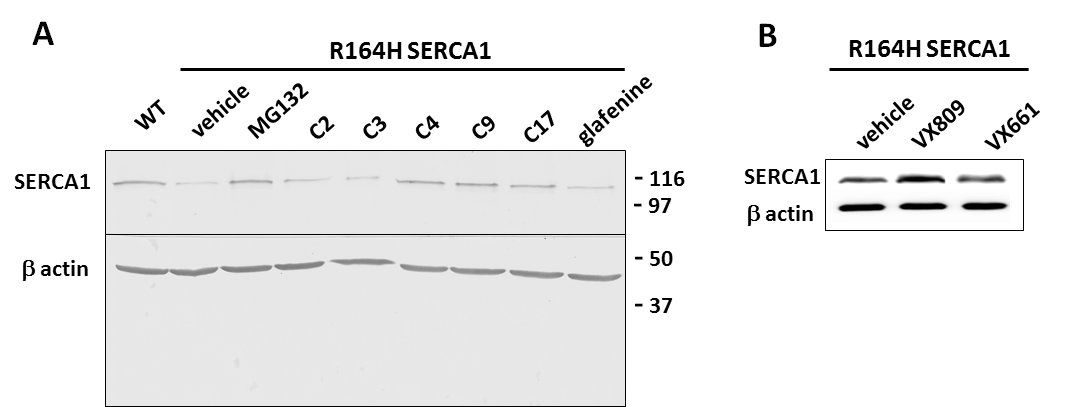


**Supplemental Figure S2.**

**Treatment with C17 CFTR corrector of Semimembranosus muscle of case 1 (A, B) and case 2 (C) PMT affected calves.** The region of the Semimembranosus and Semitendinosus muscles underwent trichotomy before beginning the pharmacological treatment. The sites of treatment were marked with a permanent marker to allow to perform the injection always in the same point.


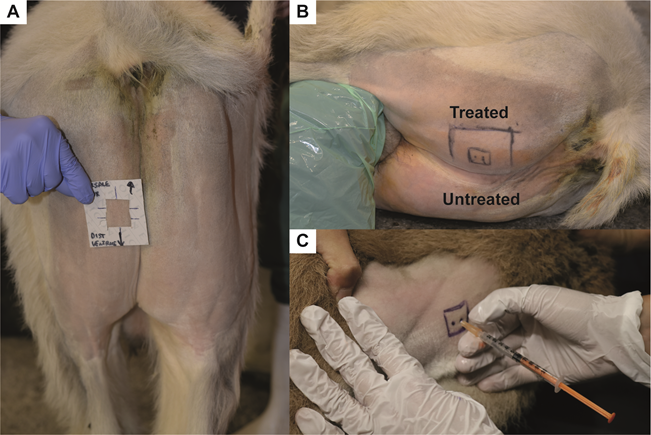


**Supplemental Figure S3.**

**Histological staining of skeletal muscles from bovine PMT- affected case 1**. Semimembranosus (**A, B**) and Quadriceps (**C, D**) muscle samples were collected after a severe contractural crisis. Transversal (**A, B, D**) and longitudinal (**C**) sections were stained with Hematoxylin and Eosin (H&E). Scale bar 50 μm.


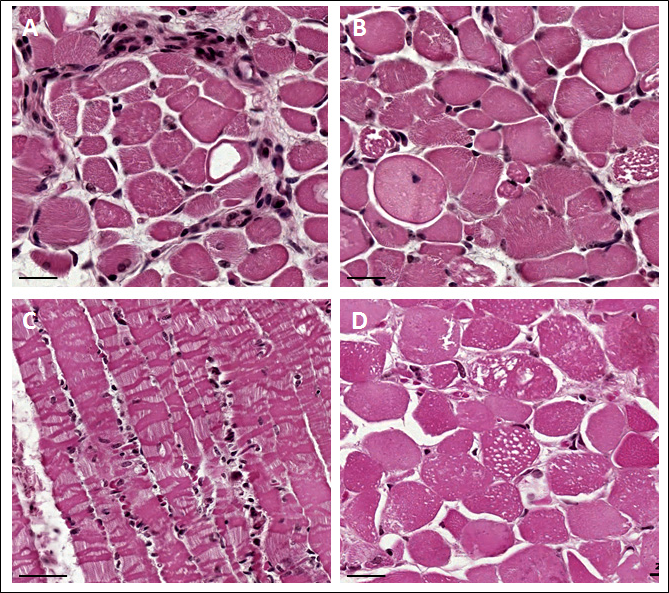


**Supplemental Figure S4.**

**Cell viability assays in HepG2 cells treated with C17 corrector at different concentrations.** Dose-response curves of C17 in HepG2 cells after 24 and 48 hrs of incubation. Data are expressed as the mean viability rate (%) (+ SD) of three biological replicates (n = 3 independent experiment), each performed in sextuplicate.


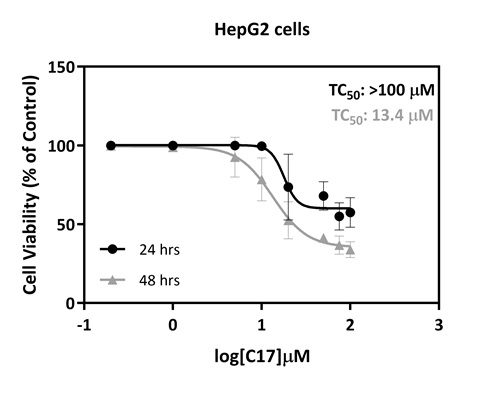


**Supplemental Figure S5.**

**Dose dependent effect of the C17 corrector on** **the rescue of R164H mutant SERCA1 in HEK293 cells.** HEK293 cells transfected with WT and R164H mutated SERCA1 cDNAs were treated with CFTR corrector C17 at concentrations 2, 4, 8 µM, or its the vehicle (veh.) DMSO (0.1%). An equal quantity of protein from total cell lysates was separated by SDS-PAGE and subjected to immunoblot analysis with antibodies specific to SERCA1 and 43 kDa beta actin, used as loading control. The graph shows the average values (+ SD) of SERCA1 expression determined by densitometric analyses of four biological replicates (n = 4 independent experiments). Values are expressed as percentage of the SERCA protein content present in cells expressing the wild type form. Statistical analysis was performed by One-way ANOVA test, multiple comparisons Dunnett test; ****, P≤ 0.0001.


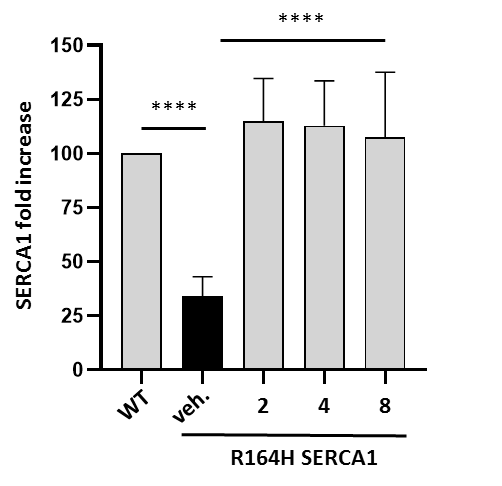

Supplement: Supplemental_Data_HMG-2025-OA_00282_Aky_rek_ddaf142 [file supplemental_data_hmg-2025-oa_00282_aky_rek_ddaf142.docx]
